# Expression of multiple *Sox* genes through embryonic development in the ctenophore *Mnemiopsis leidyi* is spatially restricted to zones of cell proliferation

**DOI:** 10.1186/2041-9139-5-15

**Published:** 2014-04-24

**Authors:** Christine E Schnitzler, David K Simmons, Kevin Pang, Mark Q Martindale, Andreas D Baxevanis

**Affiliations:** 1 Genome Technology Branch, National Human Genome Research Institute, National Institutes of Health, Bethesda, MD, USA; 2 Whitney Laboratory for Marine Bioscience, University of Florida, St. Augustine, FL, USA; 3 Sars International Centre for Marine Molecular Biology, University of Bergen, Bergen, Norway

**Keywords:** Sox, Ctenophore, Lobate, *Mnemiopsis leidyi*, Cell proliferation, Stem cell

## Abstract

**Background:**

The *Sox* genes, a family of transcription factors characterized by the presence of a high mobility group (HMG) box domain, are among the central groups of developmental regulators in the animal kingdom. They are indispensable in progenitor cell fate determination, and various *Sox* family members are involved in managing the critical balance between stem cells and differentiating cells. There are 20 mammalian *Sox* genes that are divided into five major groups (B, C, D, E, and F). True *Sox* genes have been identified in all animal lineages but not outside Metazoa, indicating that this gene family arose at the origin of the animals. Whole-genome sequencing of the lobate ctenophore *Mnemiopsis leidyi* allowed us to examine the full complement and expression of the *Sox* gene family in this early-branching animal lineage.

**Results:**

Our phylogenetic analyses of the *Sox* gene family were generally in agreement with previous studies and placed five of the six *Mnemiopsis Sox* genes into one of the major *Sox* groups: SoxB (MleSox1), SoxC (MleSox2), SoxE (MleSox3, MleSox4), and SoxF (MleSox5), with one unclassified gene (MleSox6). We investigated the expression of five out of six *Mnemiopsis Sox* genes during early development. Expression patterns determined through *in situ* hybridization generally revealed spatially restricted *Sox* expression patterns in somatic cells within zones of cell proliferation, as determined by EdU staining. These zones were located in the apical sense organ, upper tentacle bulbs, and developing comb rows in *Mnemiopsis*, and coincide with similar zones identified in the cydippid ctenophore *Pleurobrachia*.

**Conclusions:**

Our results are consistent with the established role of multiple *Sox* genes in the maintenance of stem cell pools. Both similarities and differences in juvenile cydippid stage expression patterns between *Mnemiopsis Sox* genes and their orthologs from *Pleurobrachia* highlight the importance of using multiple species to characterize the evolution of development within a given phylum. In light of recent phylogenetic evidence that Ctenophora is the earliest-branching animal lineage, our results are consistent with the hypothesis that the ancient primary function of *Sox* family genes was to regulate the maintenance of stem cells and function in cell fate determination.

## Background

*Sox* genes are among the main groups of transcription factors that regulate animal development. In general, they help specify the germline, maintain stem cells, and generate numerous cell and tissue types. In mammals and classic invertebrate model species, *Sox* genes play a fundamental role in generating neurons, heart tissue, blood vessels, and cartilage [1,2]. There are 20 *Sox* genes in vertebrates, classified into five major groups (B, C, D, E, and F) [3]. Many *Sox* genes are associated with the developing nervous system, including 12 of the 20 vertebrate *Sox* genes [4]. These transcription factors have also been implicated in human disease, specifically cancer [5,6]. *Sox* genes regulate the transcription of target genes by partnering with various proteins through diverse mechanisms [7,8] and specific *Sox* gene binding targets are continually being discovered [9].

Phylogenetic analyses have demonstrated a surprising diversity of *Sox* genes in the non-bilaterian animal lineages (ctenophores, sponges, placozoans, and cnidarians). Current thought holds that the *Sox* family first arose in the ancestor to all animals [10], then diversified into three or four groups (B, C, E, and/or F) in sponges [11,12] and four groups (B, C, E, and F) in ctenophores [13,14], the two lineages most distantly related to Bilateria [15-17]. Understanding the functions of *Sox* transcription factors in ctenophores will give insight to the roles *Sox* genes have played in the evolution of multicellularity and transcriptional gene regulatory networks.

While ctenophores (comb jellies) may appear to resemble medusae (jellyfish), which are members of the phylum Cnidaria, they exhibit complex internal and external morphology that differs drastically from that of any cnidarian (or any other animal, for that matter) [18]. External features of the animal include a mouth at one end (oral pole) and an aboral sensory complex, flanked by two anal pores, at the opposite end (aboral pole). Their bodies are comprised of an outer epidermal layer and an inner gastrodermal layer separated by mesoglea. Ctenophores are named for their eight longitudinal rows of comb plate cilia, which are used for locomotion and predation. Numerous cilia in each individual comb plate are laterally connected to form a stiff paddle-like plate, which are arranged in stacks along each comb row [19].

The aboral sensory complex includes an apical sense organ surrounded by two elongated ciliated areas known as polar fields. The apical sense organ is made up of ciliated epithelial cells and can detect changes in gravity due to four balancers that are connected to a statolith. There are four small groups of neural cells in the floor of the apical sense organ, termed ‘lamellate bodies’ [20], presumed to be photoreceptors based on morphology [20,21]. In *Mnemiopsis*, these cells express a functional opsin gene, suggesting a light-sensing role for these structures [22]. The apical sense organ also controls comb row function via a connection of each balancer to a pair of comb rows [23].

In addition to the aboral sensory complex, ctenophores have a well-developed and unique nervous system made up of a subepithelial polygonal nerve net organized as short nerve cords that extend into the tentacles [24], and a mesogleal nerve net comprised of neurons that extend through the mesoglea [24]. Ctenophores have a muscular system that spans the body wall, pharynx, and tentacles. In addition, they possess eight meridional canals, located directly beneath each of the comb rows, containing pairs of gonads (male and female in the same individual, with most species being hermaphroditic). Ovaries and testes can be distinguished by their location within the canal walls and by their small nuclear size [25,26]. Bioluminescent light-producing cells called photocytes, which also likely function in opsin-mediated light reception [22], are found in the meridional canals as well.

In terms of embryogenesis, fate-mapping experiments [27] have shown that fertilized eggs go through a highly stereotyped ctenophore-specific cleavage program in which the fate of some (but not all) blastomeres are determined at the time of their birth. Nearly all ctenophores display direct development, with embryos from pelagic ctenophores rapidly developing into a juvenile adult with a free-swimming cydippid stage in approximately 24 to 48 h [28,29]. *Mnemiopsis* cydippids measure 250 to 300 μm in diameter at hatching, around 24 h after being spawned [30]. Major adult structures are generated by multiple cell lineages, although it has not yet been possible to follow labeled embryos long enough to determine the precise origin of germ cells [27]. Germ cells are first identified in ctenophores sometime after embryos hatch out of their egg envelope as cydippids around 24 hours post fertilization (hpf); these cells are co-located with the meridional canals that give rise to the ctene rows. Multiple ovaries and testes develop on opposite sides within the meridional canals.

There are as many as 150 described species of ctenophores (along with many more undescribed species) exhibiting coastal, oceanic, and benthic lifestyles. Coastal lobate ctenophores, including *Mnemiopsis*, exhibit two expandable lobes that function as prey capture surfaces via specialized sticky colloblast cells, together with short tentacles that remain inside the lobes. In contrast, coastal cydippid ctenophores such as *Pleurobrachia* are round or oblong in shape, usually smaller than lobate ctenophores, and typically have two long branched tentacles covered with colloblasts for prey capture.

Multiple body regions are known to provide stem cell/progenitor cell pools for various cell types in ctenophores [31]. One major stem cell region in ctenophores that has been well-studied is located in the basal portion of the tentacle (the tentacle root). This region supplies multiple cell types to the growing tentacle; new colloblasts and other epidermal cells are derived from an area located along a pair of lateral ridges on the tentacle root surface [32]. An additional cell lineage in the tentacle root, located in a median ridge, gives rise to non-epithelial muscle cells and nerve cells of the tentacle mesoglea [18]. Other adult stem cell regions are located in the extremities of mature combs in progenitors of the comb rows known as polster cells, and in four specific patches of cells in the polar fields of the aboral sensory complex [31].

*Sox* genes have been extensively studied in the cydippid ctenophore, *Pleurobrachia pileus*[13,14]. These reports identified 13 *Sox* genes in this species, and provided juvenile cydippid and adult expression patterns and a gene tree for six of these genes, including members of the B, C, E, and F groups. No expression pattern was obtained for a ctenophore-specific gene called PpiSox4 that could not be placed into any of the well-characterized *Sox* groups. *In situ* hybridizations showed that all six *Pleurobrachia Sox* genes have some expression in body regions shared between juvenile and adult stages, but that expression in other regions is unique to each life stage [14]. The expression patterns also revealed previously unrecognized localized complexity in the ctenophore body plan in areas such as the apical sense organ and polar fields of the aboral sensory complex, the comb rows, and the tentacle root.

In this study, we focus on the complement and expression patterns of *Sox* genes from the lobate ctenophore *Mnemiopsis leidyi*, with particular focus on comparisons with the cydippid ctenophore *Pleurobrachia pileus*. This characterization provides further understanding of *Sox* diversity and function in ctenophores, including *Sox* expression patterns during early developmental stages, highlighting the power of studying multiple representative species from phylogenetically important taxonomic groups - as well as multiple developmental stages - to elucidate how this central group of transcription factors and their functions evolved in the earliest ancestors of extant animals.

## Methods

### Genomic survey for *Mnemiopsis Sox* genes

Recently, the whole genome sequence for *Mnemiopsis leidyi* was published and became publicly available [17]. *Sox* genes from non-bilaterian species and human were used in TBLASTN and BLASTP searches of the genome assembly, gene models, and protein models (version 2.2) of the *Mnemiopsis leidyi* genome, which are available through the *Mnemiopsis* Genome Project Portal (http://research.nhgri.nih.gov/mnemiopsis). We retrieved seven putative *Sox* sequences from these searches. After verifying the sequences via RACE-PCR (see ‘Animal collection and *in situ* hybridization’), the sequences were deposited in GenBank (Accession Numbers KJ173818-KJ173824). In some cases, the final deposited sequence differed from the predicted gene model. Here is a list of how the gene model IDs correspond to the deposited MleSox gene sequences: MleSox1 (KJ173818) = ML047927; MleSox2 (KJ173819) = ML234028; MleSox3 (KJ173820) = ML042722; MleSox4 (KJ173821) = ML06932; MleSox5 (KJ173822) = ML23337; MleSox6 (KJ173823) = ML01787; MleHMG-box (KJ173824) = ML040423.

### Phylogenetic analysis

The dataset was compiled using the available *Sox* gene complement from all non-bilaterian species plus selected bilaterian species. Anthozoan cnidarians were represented by the set of 14 *Sox* genes from the sea anemone *Nematostella vectensis*[33] and six additional published sequences from the coral *Acropora millepora*[34]. We added a set of 14 *Sox* genes from the hydrozoan cnidarian *Hydra magnipapillata* and 10 *Sox* genes from *Clytia hemisphaerica* that were previously described [35]. For sponges, we included four sequences from the demosponge *Amphimedon queenslandica*[11] and three from the demosponge *Ephydatia muelleri*, plus seven from the calcareous sponge *Sycon ciliatum*[12]. *Sox* homologs from non-bilaterian and bilaterian species were used in TBLASTN and BLASTP searches of available genome assemblies and predicted gene models of non-animal eukaryotic phyla, specifically the choanoflagellates *Monosiga brevicollis* and *Salpingoeca rosetta*. The filtered protein models for *Monosiga* v 1.0 [36] were downloaded from the Joint Genome Institute genome website. Gene models for *S. rosetta* were downloaded from the Origins of Multicellularity Sequencing Project at the Broad Institute (https://www.broadinstitute.org/annotation/genome/multicellularity_project/). A set of non-*Sox* HMG domains from the *Tcf/Lef* family was used as an outgroup. The 79 amino acid HMG-box domains of the seven putative *Mnemiopsis Sox* genes, two *M. brevicollis Sox*-like genes, and two *S. rosetta* Sox-like genes were aligned to known *Sox* homologs automatically using MUSCLE [37]. This alignment was used to perform preliminary phylogenetic analyses. Final analyses were done on an alignment that did not include the *M. brevicollis Sox*-like, *S. rosetta Sox*-like, or MleHMG-box sequences (Additional file 1). The only missing data were 11 N-terminal amino acids from the HMG-box for the following sequences: CheSox1, EmuSox1-3, and PpiSox2, PpiSox3, and PpiSox12. The tree was based on 136 HMG-box sequences. A second alignment was constructed without the *Hydra* sequences and was used to examine the effects these sequences had on the overall tree topology.

**Figure 1:**
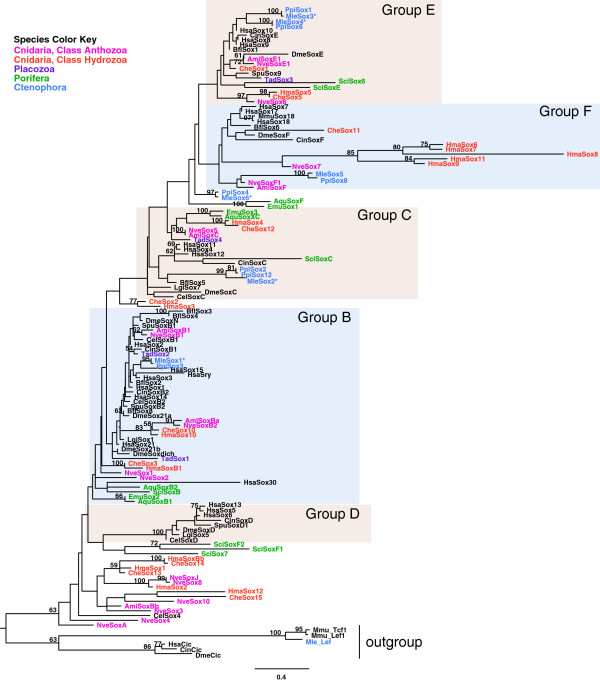
**Phylogenetic analysis of *Sox *HMG domains.** The ML tree was computed from an amino acid alignment of complete HMG domain sequences (79 amino acids in length, except for CheSox1, PpiSox2, PpiSox3, PpiSox12, EmuSox1, EmuSox2, and EmuSox3, for which only the 68 C-terminal amino acids were included). The tree likelihood was logL = -8361.8709. Numbers associated with branches correspond to ML bootstraps (100 replicates). Species names are abbreviated as follows: Ami, *Acropora millepora*; Aqu, *Amphimedon queenslandica*; Bfl, *Branchiostoma floridae*; Cel, *Caenorhabditis elegans*; Cin, *Ciona intestinalis*; Che, *Clytia hemisphaerica*; Dme, *Drosophila melanogaster*; Emu, *Ephydatia muelleri*; Hma, *Hydra magnipapillata*; Hsa, *Homo sapiens*; Lgi, *Lottia gigantea*; Mle, *Mnemiopsis leidyi*; Mmu, *Mus musculus*; Nve, *Nematostella vectensis*; Ppi, *Pleurobrachia pileus*; Sci, *Sycon ciliatum*; Spu, *Strongylocentrotus purpuratus*; Tad, *Trichoplax adhaerens*. Genes from *M. leidyi* that gave expression patterns for this study are indicated with an asterisk. Anthozoan cnidarian sequences are indicated in pink, hydrozoan cnidarian sequences are in orange, placozoan sequences are in purple, poriferan sequences are in green, ctenophoran sequences are in blue, and bilaterian sequences are in black.

To choose the best-fit model of protein evolution, we used the program ProtTest v2.4 to apply a variety of possible substitution matrices and rate assumptions [38]. The results from this indicated that the best model for the alignment was LG + Γ, where ‘LG’ indicates the substitution matrix [39], and ‘Γ’ specifies gamma-distributed rates across sites. Maximum likelihood analyses were performed with the MPI version of RAxML v7.2.8 (RAXML-HPC-MPI) [40]. We conducted four independent searches with a total of 235 randomized maximum parsimony starting trees and then compared the likelihood values among all result trees. For complex datasets, it is often necessary to perform multiple search replicates to find the same best tree multiple times to provide confidence that the tree topology with the best likelihood has been found. We found this to be the case with this dataset. One hundred bootstrapped trees were computed and applied to the best result tree. ML bootstrap values are indicated on the ML tree (Figure 1).

Bayesian analyses were performed with MrBayes3.2 [41]. MrBayes does not support the LG model of protein evolution, so we used the second best fit model from ProtTest (RtRev + Γ). Initially, we did two independent five million generation runs of five chains each, with trees sampled every 100 generations. We found that using these parameters, the ‘Average standard deviation of split frequencies’ between the two runs was 0.0148. This diagnostic value should approach zero as the two runs converge and an average standard deviation below 0.01 is a very good indication of convergence, while any value between 0.01 and 0.05 is considered acceptable for convergence. We then did two independent five million generation runs of nine chains each, with trees sampled every 100 generations and a heating parameter of 0.05 (default heating is 0.2) and achieved an average standard deviation of split frequencies of 0.0101. We also ran MrBayes with the ‘mixed’ amino acid model option (prset aamodelpr = mixed) using the same parameters and found no difference in the convergence diagnostic value or in the resulting tree compared to the tree generated with the RtRev + Γ model. Additional convergence diagnostics, examined with the help of AWTY [42], indicated a conservative burn-in of 0.25. The runs reached stationarity, and adjusting the burn-in did not affect the topology. A majority rule consensus of 37,500 trees was produced and posterior probabilities were calculated from this consensus. Trees were rerooted in FigTree v1.3.1 [43]. Bayesian posterior probabilities are shown on the Bayesian tree (Additional file 2: Figure S1).

### Animal collection and *in situ* hybridization

*Mnemiopsis leidyi* adults were collected from Eel Pond or the NOAA Rock Jetty, Woods Hole, MA, USA, during the months of June and July and spawned as previously described [44]. RNA was extracted from embryos with TRI Reagent (Molecular Research Center, Cincinnati, OH, USA) and reverse transcribed to generate cDNA (SMART RACE cDNA Amplification Kit, Clontech Laboratories, Inc., Mountain View, CA, USA). This cDNA was used as a template to isolate the genes of interest. Individual RACE-PCR products were cloned and sequenced, and sequences were aligned to the genomic sequence.

For whole-mount *in situ* hybridization, embryos were fixed at various stages from freshly collected nucleated embryos (0 hpf) to newly hatched cydippids (24 hpf). They were stored in methanol at -20°C until used. Sequences, ranging in length from 650 to 2,000 bp, were used to transcribe digoxigenin-labeled RNA probes (Ambion/Applied Biosystems, Austin, TX, USA). These probes were hybridized for 48 h at 60°C and detected using an alkaline phosphatase-conjugated digoxigenin antibody (Roche Applied Science, Indianapolis, IN, USA), and the substrates nitro-blue tetrazolium (NBT)/5-Bromo-4-chloro-3-indolyl phosphate (BCIP). After detection, specimens were washed with phosphate-buffered saline (PBS) and transferred through a glycerol series up to 70% glycerol. They were then mounted, viewed under a compound microscope (Zeiss AxioSkop 2), and imaged using a digital imaging system (AxioCam HRc with Axiovision software, Zeiss, Thornwood, NY, USA). Color balance and brightness were adjusted using Photoshop software (Adobe Systems Incorporated, San Jose, CA, USA). Additional details of the *in situ* hybridization protocol for *Mnemiopsis* have been previously described [44]. All *in situ* images presented here are available online via the comparative gene expression database, Kahikai (http://www.kahikai.com).

### Cell proliferation labeling with EdU

EdU (ethynyl deoxyuridine) is a uridine analog similar to BrdU. To measure cell proliferation, cydippids were fixed and processed for fluorescent detection of incorporated EdU using the Click-iT EdU labeling kit (Invitrogen, Carlsbad, CA, USA), which incorporates EdU in cells that are undergoing the S phase of mitosis. Specifically, cydippids aged 18 to 24 h were incubated in EdU labeling solution for 15 to 20 min and then fixed using 4% paraformaldehyde with 0.02% glutaraldehyde for 30 min. After three washes in PBS, they were stored in PBS at 4°C until subsequent use. Prior to the Click-iT reaction, cydippids were washed for 20 min in PBS plus 0.2% Triton. The Click-iT reaction was performed according to manufacturer instructions, using the Alexa-488 reaction kit. To visualize nuclei, cydippids were also stained with Hoechst 33342 (Invitrogen, Molecular Probes). Cydippids were mounted in PBS, examined, and imaged under a Zeiss Axio Imager or LSM710 confocal microscope.

## Results

### Phylogenetic relationships and classification of *Mnemiopsis Sox* genes

We identified six members of the *Sox* family from the *Mnemiopsis leidyi* genome, all with complete HMG-box domains. A seventh sequence with an HMG-box domain (MleHMG-box) did not fall within the *Sox* gene family in our preliminary phylogenetic analyses and was excluded from our final alignments and trees. Phylogenetic analyses of the six *Mnemiopsis Sox* sequences, combined with all previously published non-bilaterian *Sox* sequences and several representative bilaterian *Sox* sequences, constructed the metazoan-specific *Sox* family phylogeny, including the major known groups (B, C, D, E, and F; Figure 1). From this analysis, five *Mnemiopsis Sox* genes were classified into four groups (B, C, E, and F), with an additional gene (MleSox6) branching at the base of the E and F groups (Figure 1). According to the tree reconstruction, MleSox1 belongs to group B, MleSox2 belongs to group C, MleSox3 and MleSox4 branch within group E, and MleSox5 is found within group F. Each of the *Mnemiopsis Sox* genes has a clear ortholog in the ctenophore *Pleurobrachia*, although the two SoxC genes PpiSox2 and PpiSox12 seem to be the result of a lineage-specific duplication within *Pleurobrachia* and MleSox2 is sister to these two sequences.

### Phylogenetic relationships and classification of non-bilaterian *Sox* genes

As observed in other recent studies [12,35], a number of the non-bilaterian *Sox* sequences could not be classified into any of the previously identified major *Sox* groups (Figure 1), including two ctenophore sequences (MleSox6 and PpiSox4) and two sponge sequences (AquSoxF and EmuSox1) that branch at the base of the E and F groups. Several cnidarian *Sox* sequences from various species (*Acropora millepora*, *Clytia hemisphaerica*, *Hydra magnipapillata*, and *Nematostella vectensis*) also could not be classified into the traditional groups, including a group of 14 cnidarian sequences that fall within their own clade in the *Sox* family (Figure 1). This group includes the nematode CelSox4 gene. To test the possible effects of long-branch attraction due to inclusion of some of the *Hydra* sequences, we constructed separate trees that did not include any *Hydra Sox* sequences, but found the same overall tree with only minor rearrangements of branches (data not shown). As noted in other phylogenetic analyses of the *Sox* HMG-box [13,35], low statistical support of the major clades likely stems from the short sequence length used for the analyses and the inclusion of a large number of taxa sampled across a wide evolutionary distance.

### Comparison of *Sox* phylogeny with previous studies

The trees generated from the maximum likelihood (ML; Figure 1) and Bayesian (Additional file 2: Figure S1) analyses have the same overall topology; there are only a few individual branches that differ between the two trees (specifically, HsaSox30, CheSox2, HmaSox3, TadSox1, SciSox6, and SciSoxE). Overall, our trees (Figure 1; Additional file 2: Figure S1) were in general agreement with other recent surveys of non-bilaterian *Sox* genes [12,35], with a few notable exceptions, denoted in bold text in Table 1. A previous analysis of the *Sox* complement from the calcareous sponge *Sycon* placed SciSoxE in the SoxE group, SciSoxF1 and SciSoxF2 in the SoxF group, and was unable to classify two other genes (SciSox6 and SciSox7) into any known group [12]. In contrast, our Bayesian analyses consistently place SciSoxE in an unclassified position at the base of the SoxE and SoxF groups, while our ML analyses place it in the SoxE group, calling into question whether sponges have a clear SoxE homolog. Neither of our analyses placed any sponge sequence in the SoxF group. Three *Sycon* genes (SciSox7, SciSoxF1, and SciSoxF2) branch next to the exclusively bilaterian SoxD group in both of our analyses, albeit with low ML bootstrap support and a low Bayesian posterior probability. The branch uniting the *Sycon* sequences that places them next to the SoxD clade was unstable in both of our analyses, based on post-tree analysis using PhyUtility [45], a program that calculates branch attachment frequency and leaf stability metrics. Therefore, it is unclear whether these genes are truly related to SoxD genes, whether this was an artifact of tree reconstruction methods, or whether this may be due to possible sequence convergence. Looking across all result trees from all of our analyses, we see that the *Sycon* sequences previously classified as SoxF (SciSoxF1 and SciSoxF2) occur together in a position either next to the SoxD group (as seen in Figure 1) or in an unclassified position at the base of the tree in over 90% of trees. Fewer than 10% of our result trees place these two sequences in an unclassified position at the base of the SoxE and SoxF groups together with AquSoxF and EmuSox1. We did not observe the placement of these *Sycon* sequences in any known group in any of our result trees, regardless of the tree construction method or sequences included.

**Table 1:** **Classification of *Sox *** **genes from this study**

	**Bilaterian animal**	**Non-bilaterian animals**									**Unicellular filozoans**	
	**Deuterostomia**	**Cnidaria**				**Placozoa**	**Porifera**		**Ctenophora**		**Choanoflagellata**	
***Sox *** **group**	***Homo sapiens***	***Nematostella***	***Acropora***	***Hydra***	***Clytia***	***Trichoplax***	***Amphimedon***	***Sycon***	***Mnemiopsis***	***Pleurobrachia***	***Monosiga***	***Salpingoeca***
**B group**	HsaSry	NveSox1*	AmiSoxB1*	HmaSoxB1	CheSox3*	TadSox1^c^	AquSoxB1	SciSoxB*	MleSox1*	PpiSox3*^d^		
	HsaSox1	NveSox2*	AmiSoxBa*	HmaSox10	CheSox10*	TadSox2	AquSoxB2			PpiSox5^a^		
	HsaSox2	NveSoxB1*								PpiSox7^a^		
	HsaSox3	NveSoxB2*								PpiSox9^a^		
	HsaSox14									PpiSox10^a^		
	HsaSox15									PpiSox11^a^		
	HsaSox21											
	HsaSox30^c^											
**C group**	HsaSox4	NveSox5*	AmiSoxC*	HmaSox4	CheSox12*	TadSox4	AquSoxC	SciSoxC*	MleSox2*	PpiSox2*^d^		
	HsaSox11									PpiSox12*^d^		
	HsaSox12									PpiSox13^a^		
**D group**	HsaSox5											
	HsaSox6											
	HsaSox13											
**E group**	HsaSox8	NveSoxE1*	AmiSoxE1*	HmaSox5	CheSox1*^d^	TadSox3		**SciSox6**^**c**^	MleSox3*	PpiSox1*		
	HsaSox9	NveSox6			CheSox5*			SciSoxE*^c^	MleSox4*	PpiSox6*		
	HsaSox10											
**F group**	HsaSox7	NveSoxF1*	AmiSoxF*	**HmaSox6**	CheSox11*				MleSox5	PpiSox8*		
	HsaSox17	NveSox7		**HmaSox7**								
	HsaSox18			**HmaSox8**								
				**HmaSox9**								
				**HmaSox11**								
**Unclassified**		NveSoxA	AmiSoxBb*	**HmaSox1**	CheSox2*^c^		AquSoxF	SciSox7*	MleSox6*	PpiSox4	MbrSox-like1^b^	SroSox-like1^b^
		NveSox3*		HmaSox2	CheSox13*			SciSoxF1*	MleHMG-box^b^		MbrSox-like2^b^	SroSry-like1^b^
		NveSox4		HmaSox3^c^	CheSox14*			SciSoxF2*				
		NveSox8		HmaSox12								
		NveSox10		**HmaSoxBb**	CheSox15*							
		NveSoxJ										
**Total # *Sox *** **genes/groups**	**20/5**	**15/4**	**6/4**	**14/4**	**10/4**	**4/3**	**4/2**	**7/3**	**6/4**	**13/4**	**0/0**	**0/0**

An asterisk denotes genes with published *in situ* expression patterns. Gene names in bold text were previously classified differently [12,35].

^a^ Not represented in trees because only short partial sequence is available (20 aa missing from HMG-box).

^b^ Not represented in final trees or counted in the total as preliminary analyses indicated that these are not likely to be true *Sox* family genes.

^c^ Classification sensitive to tree search method used in this study, with classification from ML analysis shown.

^d^ Partial sequence (11 aa missing from 5′ end of HMG-box).

In our trees, a cluster of five paralogous *Hydra Sox* genes are located in the SoxF group, while previous analyses concluded that the *Sox*F group had apparently been lost from this lineage [35]. This placement was consistent across all of our result trees, regardless of the tree construction method or the sequences included. The *Clytia Sox* study [35] placed four hydrozoan *Sox* sequences (CheSox13, CheSox14, HmaSox1, and HmaSoxBb) and two anthozoan sequences (NveSox3 and AmiSox3) in the SoxB group, while in our trees, these sequences consistently fell in the unclassified group of 14 cnidarian sequences plus the nematode CelSox4. We have summarized our classification of all non-bilaterian *Sox* genes based on our ML analysis in Table 1.

A previous study identified a putative *Sox* gene from the choanoflagellate *Monosiga brevicollis*[36]. We identified two *Sox*-like sequences from the *M. brevicollis* genome (Joint Genome Institute ID: 12602, 12133), as well as two *Sox*-like sequences from the genome of another choanoflagellate, *Salpingoeca rosetta* (Broad Institute ID: PTSG_01623.1, PTSG_02101.1). In our preliminary analyses, however, these sequences, together with the *Mnemiopsis* MleHMG-box gene, always clustered together outside the *Sox* gene family with outgroup sequences, suggesting that they are not true *Sox* genes. We excluded these sequences from our final alignments and trees but include them in Table 1. Our result is in agreement with a recent in-depth study of transcription factors in the genome of the unicellular holozoan *Capsaspora owczarzaki* and its close relatives [10]. In that study, the authors found that HMG-box transcription factors arose early in eukaryotic evolution, followed by ‘*Sox*-like’ HMG-box genes, which arose in the ancestor to choanoflagellates (after the lineage leading to *C. owczarzaki* diverged), followed by the evolution of *Sox* and *Tcf/Lef* HMG-box families at the base of the animals. Further study of the choanoflagellate and ctenophore ‘*Sox*-like’ sequences will help to clarify the origin and possible functions of these genes.

Two ctenophore *Sox* sequences (MleSox1 and PpiSox3) fall into group B, within a subclade of exclusively bilaterian *Sox* sequences that includes three human paralogs (HsaSox15, HsaSry, and HsaSox3). Jager et al. [35] pointed out a similar placement of the PpiSox3 gene in their *Sox* phylogeny and highlighted the evolutionary implications of this placement, including the possibility that other non-bilaterian orthologs were lost from this subclade or that the placement of the ctenophore *Sox* group B sequences in this position may be an artifact of tree reconstruction methods or due to possible sequence convergence.

Within group C, there is a non-bilaterian clade consisting of sponge, cnidarian, and placozoan sequences. Three ctenophore SoxC sequences (MleSox2, PpiSox2, and PpiSox12) form a cluster with a sequence from the chordate *Branchiostoma floridae* (BflSox5) that falls next to a cluster with three human sequences (HsaSox4, HsaSox11, and HsaSox12), one sequence from *Ciona intestinalis* (CinSoxC), and one sponge sequence (SciSoxC).

A bilaterian SoxF subgroup was recovered in all analyses and included a single non-bilaterian member, CheSox11 from *Clytia*. Two sister subgroups within the overall SoxF group contain the remaining non-bilaterian sequences. One subgroup has a cluster of five *Hydra* sequences and a single *Nematostella* sequence (NveSox7). The other subgroup includes two ctenophore sequences (MleSox5 and PpiSox8), a *Nematostella* sequence (NveSoxF1), and AmiSoxF from *Acropora*.

Group E *Sox* genes include a subgroup of four ctenophore sequences (a set of paralogs from *Mnemiopsis*, MleSox3 and MleSox4; and another set from *Pleurobrachia*, PpiSox1 and PpiSox6). This subgroup is found within a larger group of bilaterian and non-bilaterian SoxE sequences. A branch with two unclassified ctenophore *Sox* sequences (MleSox6 and PpiSox4) falls at the base of Group E and Group F (Figure 1). In a previous study [35], PpiSox4 was located in the same unclassified position.

### *Mnemiopsis* SoxB gene expression

**Figure 2:**
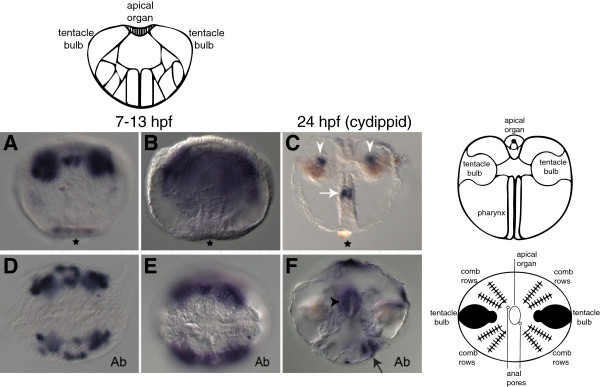
**Expression patterns of MleSox1 during development.** The schematic at the top depicts the stage of development directly underneath (7 to 13 hpf lateral view), while the schematics along the side depict the stage directly adjacent (24 hpf lateral view on top; 24 hpf aboral view on bottom), identifying some of the major features and structures (redrawn from [46]). Panels **A-C** are lateral views, while panels **D-F** are aboral views (denoted by ‘Ab’). **(A, B, D, E)***In situ* hybridization of embryos 7 to 13 hpf. **(C, F)***In situ* hybridization of cydippids 24 hpf. **(C)** MleSox1 expression in the upper tentacle bulbs (white arrowheads), and pharynx (white arrow). **(F)** MleSox1 expression in the apical organ (black arrowhead), and in the uppermost part of at least one set of comb rows (black arrow).

Expression of MleSox1, a member of the SoxB group, was not detected by *in situ* hybridization before or immediately after gastrulation (which occurs around 4 hpf). Light expression is seen in the developing embryo around 7 hpf, around the blastopore, in cells that invaginate to form the pharynx in the cydippid (Figures 2A and B). Expression in a patch of cells in the pharynx can later be seen in the cydippid (Figures 2C). Expression at 7 to 13 hpf is also found in epidermal cells that later form the comb plates (Figures 2A and B); the expression of these epidermal cells expands along the body column as the embryo develops (Figures 2B and E) but then becomes very light and is restricted to the uppermost part of the comb rows in the cydippid (visible in Figure 2F but not in 2C). Under the epidermal expressing cells, expression is found in a small number of cells that later form a part of the upper tentacle bulb in the cydippid (Figures 2C). At 7 to 13 hpf, additional expression is found in three patches of ectodermal cells along the sagittal plane; the innermost patch of these cells contributes to the apical organ. In the cydippid, expression can be seen in the apical organ (Figures 2F). By comparison, PpiSox3 also exhibits expression in the pharynx, tentacle bulb, and apical sensory organ during the juvenile cydippid stage, although comb row expression is not seen [14].

### *Mnemiopsis* SoxC gene expression

**Figure 3:**
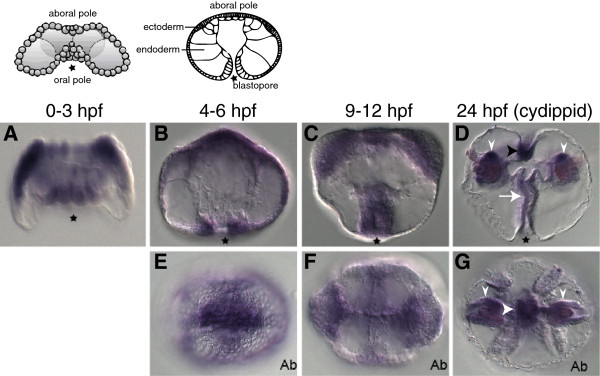
**Expression patterns of MleSox2 during development.** The schematics at the top depict the stage of development directly underneath (0 to 3 hpf lateral view, and 4 to 6 hpf lateral view; redrawn from [46]). Panels **A-D** are lateral views, while panels **E-G** are aboral views (denoted by ‘Ab’). **(A)***In situ* hybridization of an embryo 0 to 3 hpf. **(B, E)***In situ* hybridization of embryos 4 to 6 hpf. **(C, F)***In situ* hybridization of embryos 9 to 12 hpf. **(D, G)***In situ* hybridization of cydippids 24 hpf. **(D)** MleSox2 expression in the pharynx (white arrow), tentacle bulbs (white arrowheads), and the apical organ (black arrowhead). **(G)** MleSox2 expression in the tentacle bulbs (white arrowheads), and the apical organ (white arrowhead).

Expression of MleSox2, the SoxC group member, was detected ubiquitously during early cleavage stages representing maternally deposited expression (Figure 3A). Post-gastrulation (4 to 6 hpf), the expression is split between the oral and aboral halves of the developing embryo, specifically around the blastopore on the oral half, and in mesodermal and ectodermal cells on the aboral half (Figures 3B and E). Expression is ubiquitous in the pharynx and the aboral half of the embryo at 9 to 12 hpf (Figures 3C and F). In the juvenile cydippid, expression is restricted to the pharynx, tentacle bulbs, and the apical sense organ, remaining uniformly expressed in each tissue (Figures 3D and G). In juvenile cydippids from *Pleurobrachia*, PpiSox2/12 was similarly expressed in the tentacle base and apical sense organ, but not in the pharynx. PpiSox2/12 also exhibited expression in small spots within the comb rows [14].

### *Mnemiopsis* SoxE gene expression

**Figure 4:**
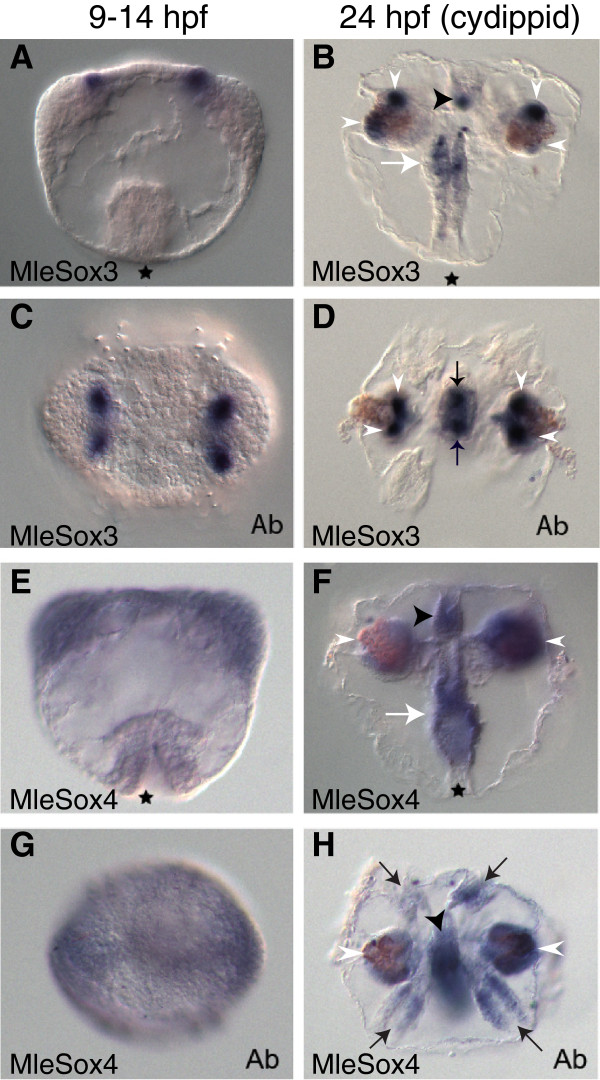
**Expression patterns of MleSox3 and MleSox4 during development.** Panels **A, B, E,** and **F** are lateral views, while panels **C, D, G,** and **H** are aboral views (denoted by ‘Ab’). **(A, C)** MleSox3 *in situ* hybridization of embryos 9 to 14 hpf. **(B, D)** MleSox3 *in situ* hybridization of cydippids 24 hpf. **(B)** MleSox3 expression in the upper pharynx (white arrow), apical organ (black arrowhead), and in four distinct regions of the upper tentacle bulbs (white arrowheads). **(D)** MleSox3 expression in four distinct regions of the upper tentacle bulbs (white arrowheads), and two main cell groups of the apical organ (black arrows). **(E, G)** MleSox4 *in situ* hybridization of embryos 9 to 14 hpf. **(F, H)** MleSox4 *in situ* hybridization of cydippids 24 hpf. **(F)** MleSox4 expression in the pharynx (white arrow), tentacle bulbs (white arrowheads), and apical organ (black arrowhead). **(H)** MleSox4 expression in the comb rows (black arrows), the tentacle bulbs (white arrowheads), and apical organ (black arrowhead).

MleSox3 is expressed during embryogenesis at 9 to 14 hpf in four groups of mesodermal cells that make up part of the upper tentacle bulb (Figures 4A and C). During the cydippid stage, expression in this region is concentrated in four distinct regions of the upper tentacle bulbs (Figures 4B and D). Additionally, MleSox3 expression is found in groups of cells in the upper pharynx, as well as in the apical sense organ in two main cell groups along the sagittal axis where the base of the apical organ connects to the polar fields (Figures 4B and D). In comparison, PpiSox1, the ortholog to MleSox3, was similarly expressed near the tentacle base, in four small spots around the pharynx, and in five spots in the apical sense organ [14].

MleSox4 expression is lightly ubiquitously expressed at 9 to 14 hpf in parts of the developing pharynx, in ectodermal and mesodermal cells that make up the tentacle apparatus, and in cells that form the apical organ (Figures 4E and G). During the juvenile cydippid stage, expression encompasses the entirety of the comb rows (Figure 4H). The ubiquitous expression found in earlier stages continues in the pharynx, the tentacle bulbs, and the apical organ of the cydippid (Figures 4F and H). In contrast, PpiSox6 expression during the juvenile cydippid stage was seen exclusively in the comb rows [14].

### Expression of MleSox6

**Figure 5:**
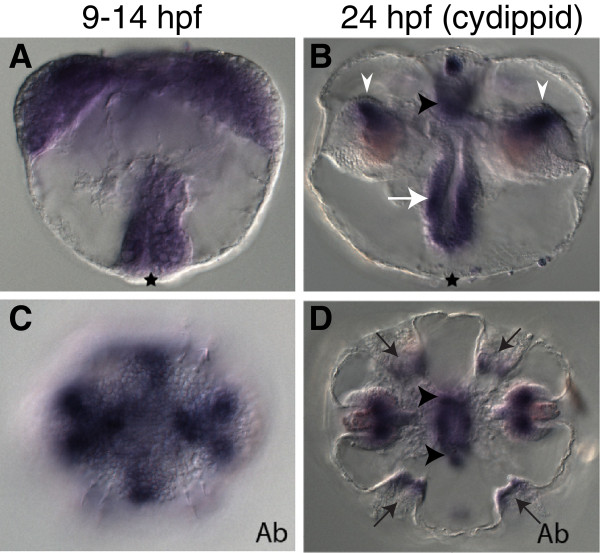
**Expression patterns of MleSox6 during development.** Panels **A and B** are lateral views, while panels **C and D** are aboral views (denoted by ‘Ab’). **(A, C)***In situ* hybridization of embryos 9 to 14 hpf. **(B, D)***In situ* hybridization of cydippids 24 hpf. **(B)** MleSox6 expression in the upper tentacle bulbs (white arrowheads), the pharynx (white arrow), and the apical organ floor (black arrowhead). **(D)** MleSox6 expression in the apical ends of the comb rows (black arrows), and the apical organ floor where it extends out towards the polar fields (black arrowheads).

MleSox6, which was unclassified by the phylogenetic analysis, is initially expressed around 9 hpf in animals with already developed and functional comb plates. At 9 to 14 hpf, expression is distributed equally throughout the pharynx and stops where the pharynx meets the endoderm; this expression continues throughout the cydippid stage (Figures 5A and B). The aboral expression at 9 to 14 hpf encompasses parts of the mesodermally and ectodermally derived portions of the tentacle bulbs (Figures 5A and C). Expression is also found in cells that later form part of the developing apical sense organ. During the cydippid stage, expression can be found towards the apical ends of the comb rows (Figure 5D). Expression of MleSox6 during the cydippid stage also encompasses the apical organ floor (Figure 5B) and extends out to the polar fields (Figure 5D). The uppermost parts of the tentacle bulbs show expression at this stage (Figure 5B), and light expression continues through mesodermally derived cells connected to the base of the apical sense organ (Figure 5B). There are no expression patterns available for the orthologous gene in *Pleurobrachia*, PpiSox4.

Despite several attempts, expression patterns were not detected for the *Mnemiopsis* SoxF group member (MleSox5) during any developmental stage. In support of this, RNA-Seq data generated for the *Mnemiopsis* genome paper [17] from mixed stage embryos (approximately 15 to 30 hpf) also do not indicate expression of this gene (data available through the *Mnemiopsis* Genome Web Portal: http://research.nhgri.nih.gov/mnemiopsis/, using the ‘CL2’ track of the Genome Browser). We also made several attempts to generate expression patterns for the *Sox*-like MleHMG-box gene, but did not detect any expression during development. Similar to MleSox5, the independent RNA-Seq evidence also suggests that this gene is not expressed in the mixed stage embryo sample mentioned above. A comparison of all *Mnemiopsis* and *Pleurobrachia* expression patterns is summarized in Table 2.

**Table 2:** **Summary of *Mnemiopsis *** **and *Pleurobrachia Sox *** **expression patterns**

		**Group B**		**Group C**		**Group E**		**Group E**		**Group F**		**Unclassified (EF)**	
		**MleSox1**	**PpiSox3**	**MleSox2**	**PpiSox2/PpiSox12**	**MleSox3**	**PpiSox1**	**MleSox4**	**PpiSox6**	**MleSox5**	**PpiSox8**	**MleSox6**	**PpiSox4**
**Early embryo**	Tentacle bulb	+	nd	+	nd	+	nd		nd	nd	nd	+	nd
	Apical sensory organ	+	nd	+	nd		nd		nd	nd	nd	+	nd
	Comb row	+	nd		nd		nd		nd	nd	nd		nd
	Pharynx	+	nd	+	nd		nd	+	nd	nd	nd	+	nd
**Juvenile cydippid**	Tentacle bulb	+*	+*	+*	+*	+*	+*	+		nd	+	+	nd
	Apical sensory organ	+*	+*	+*	+*	+*	+*	+		nd		+	nd
	Comb row	**			+			+*	+*	nd		+	nd
	Pharynx	+*	+*	+		+*	+*	+		nd		+	nd
	Stomach									nd	+	nd	nd
**Adult**	Tentacle bulb	nd	+	nd	+	nd	+	nd	+	nd	+	nd	nd
	Apical sensory organ	nd		nd	+	nd	+	nd		nd		nd	nd
	Polar fields (Z bodies)	nd	+	nd		nd		nd		nd		nd	nd
	Comb row	nd		nd	+	nd		nd	+	nd		nd	nd
	Pharynx	nd		nd		nd		nd		nd		nd	nd
	Gastrovascular canal	nd		nd		nd		nd		nd	+	nd	nd
	Gonads	nd		nd	+	nd		nd		nd		nd	nd

Orthologs are represented in pairs of columns by *Sox* group. Expression is indicated by a plus sign (+). A single asterisk (*) shows agreement in expression patterns between orthologous genes from the two ctenophore species. ‘nd’ denotes no data. The double asterisk (**) for MleSox1 expression in the comb row refers to light expression seen only in the uppermost part of the comb row at the cydippid stage.

### Cell proliferation staining of *Mnemiopsis* embryos

**Figure 6:**
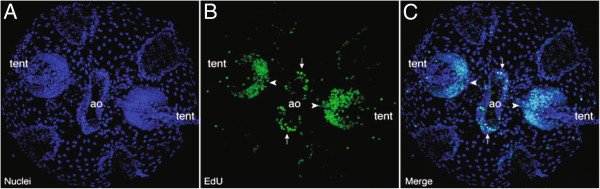
**Confocal projections of EdU incorporation experiments in *M. leidyi *cydippids 18 to 24 hpf. (A)** Hoechst 33342 stained nuclei in blue. **(B)** EdU-labeled nuclei in green, showing regions of cell proliferation, including the tentacle bulbs (‘tent’, white arrowheads) and apical organ (‘ao’, white arrows). **(C)** Merged view of A and B showing the overlap between nuclei and regions of EdU incorporation. Increased EdU labeling is present in the tentacle bulbs (white arrowheads) and the apical organ floor (white arrows), while isolated EdU-labeled nuclei can be seen in the developing comb rows.

*Mnemiopsis* juvenile cydippids (18 to 24 hpf) were labeled with EdU to identify regions of cell proliferation (Figure 6). Results show increased labeling in the tentacle bulbs and the apical sense organ, specifically in the apical organ floor (Figure 6B). There was additional labeling of individual nuclei in the developing comb rows (Figures 6B and C). There were minimal levels of labeling in the epidermis and in the pharynx (not visible in Figure 6). These results are generally consistent with regions of cell proliferation found in adult *Pleurobrachia*[31].

## Discussion

### *Sox* gene phylogeny

**Figure 7:**
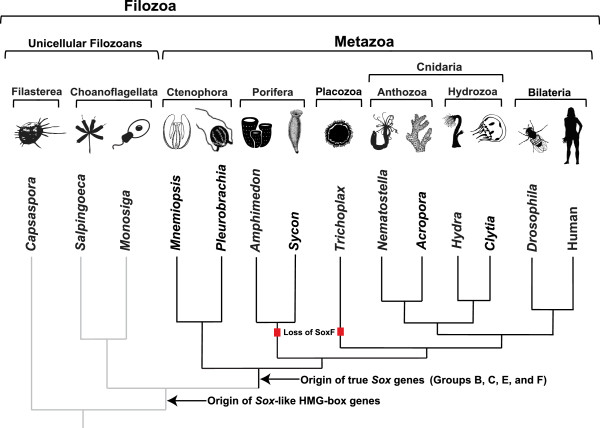
**Evolutionary history of the of *Sox *transcription factor family.** According to our analyses and within the context of the animal tree that places ctenophores as the earliest-branching group [15-17], true *Sox* genes arose at the base of the animals, while *Sox*-like HMG-box genes are present in Choanoflagellata. A red square indicates the loss of the SoxF group from that lineage. Alternative animal trees that place Porifera or Placozoa as the earliest branch would indicate that the SoxF group was absent in the earliest animal ancestor but arose in the lineage leading to Ctenophora.

A recent extensive set of phylogenetic analyses of animals and closely related non-animals that includes data from the first ctenophore genome (*Mnemiopsis*) supports a tree with Ctenophora branching before all other animal lineages [17], which is consistent with some other recent phylogenomic analyses [15,16]. While this new evidence regarding the phylogenetic placement of the ctenophores is compelling, it is worth noting that other phylogenies have been put forward, placing either Porifera [47] or Placozoa [48] as the earliest-branching animal lineage; these alternative phylogenies place Ctenophora in various locations within the non-bilaterian lineages (reviewed in [17,49]). Regardless of the branching order of the non-bilaterian animal phyla, our analyses of *Sox* family genes are consistent with the hypothesis that true *Sox* family genes arose at the base of the animals and that four major groups of *Sox* genes (B, C, E, and F) were fully diversified in ctenophores (Figure 7). This evolutionary scenario indicates that the *Sox* family of transcription factors diversified early and remained relatively stable throughout animal evolution. Overall, our trees are largely in agreement with recent studies focused on *Sox* phylogeny, with a few interesting exceptions. Neither of the sponge sequences that were placed in the SoxF group in a previous study (specifically, SciSoxF1 and SciSoxF2 [12]) were found in the SoxF group in any of our analyses. Although the position of these sequences was ‘unstable’ in our searches, none of our analyses placed them in the SoxF group. The lack of a clear SoxF gene in Porifera in our analyses raises the possibility that this *Sox* group was lost in this lineage (Figure 7); alternatively, if sponges branch before ctenophores on the animal tree, then the SoxF group may not have been present in the ancestor of all animals, first arising in the lineage leading to ctenophores. Alternately, the two or three sponge *Sox* genes that branch in an unclassified position outside of the SoxE and SoxF groups (AquSoxF and EmuSox1, also seen in this position in previous studies [13,35]; and SciSoxE, seen in this position in our Bayesian analysis only) could perhaps be members of the SoxE or F groups that have diverged over time (Figure 1).

In our ML analysis, two *Sycon Sox* sequences (SciSoxE and SciSox6) are located within the SoxE group (Figure 1); however, in our Bayesian analysis (Additional file 2: Figure S1) these two sequences are found elsewhere (SciSoxE is in an unclassified position outside the SoxE and SoxF groups, and SciSox6 is located in a subgroup next to Group D). Further, in all of our analyses, three *Sycon Sox* sequences (SciSoxF1, SciSoxF2, and SciSox7) form a poorly supported subgroup next to the bilaterian Group D *Sox* sequences. The lack of agreement about where these sponge *Sox* sequences fall may be due to differences in tree reconstruction methods and/or outgroups used. Although Fortunato et al. [12] used the same ‘LG + Γ’ model of evolution for their ML analyses, they did fewer independent ML runs with fewer starting trees for each and used a different set of outgroup sequences. Stable placement of these sponge sequences may be resolved in the future with the addition of more sponge sequences or improvements in tree reconstruction methods. Functional studies of these genes will also help elucidate how closely they align with genes in known *Sox* groups.

The other notable difference between our trees and previously published trees is the location of several *Hydra Sox* genes. In our analyses, a cluster of five *Hydra Sox* genes is clearly nested within the SoxF clade with high support. This placement was consistent across tree construction methods and datasets used, whereas previously, these sequences were found in an unclassified position outside known *Sox* groups [35]. Our results indicate that SoxF genes were not lost in the lineage leading to *Hydra*, and are present in all cnidarians surveyed to date. Overall, this suggests that non-bilaterian Group F *Sox* genes are present in all lineages except Placozoa and Porifera (Figure 7).

### General characteristics of *Mnemiopsis Sox* gene expression

We determined the expression patterns of five out of six *Mnemiopsis Sox* genes in developing embryo and juvenile cydippid stages. Expression patterns determined through *in situ* hybridization generally revealed spatially restricted *Sox* expression in somatic cells within zones of cell proliferation, as determined by EdU staining (Table 2, Figure 6). These zones were located in the developing apical sense organ, upper tentacle bulbs, and comb rows in *Mnemiopsis*, all regions previously shown to be regions of cell proliferation and/or stem cell regions in adult specimens of the ctenophore *Pleurobrachia*[31]. The experiments here show an overlap between regions of high levels of cell proliferation and regions of *Sox* gene expression, which supports the hypothesis that cells in these regions are stem cells or progenitor cells. The *Sox* genes expressed in these regions may be maintaining these cells in an undifferentiated state and/or regulating cell proliferation and renewal. The somatic expression of these genes could also play a role in the regenerative capacity of ctenophores. *Mnemiopsis* is known to be able to regenerate even when a large portion of its body is removed [50,51]. Overall, our results are consistent with the established role of multiple *Sox* genes in the maintenance of stem cell pools and as regulators of progenitor and stem cell fate [52,53].

### Comparison of *Sox* gene expression across *Mnemiopsis* developmental stages

The *Mnemiopsis Sox* mRNA expression patterns we generated follow a time course of development from early cleavage stages to the juvenile cydippid stage (approximately 24 hpf). There were no consistent patterns for all *Sox* genes within a particular developmental stage, with each *Sox* gene exhibiting its own unique pattern (Table 2).

*Sox* gene expression has been seen in germline cells in *Clytia hemisphaerica* (*Sox* groups B, C, and E), and in adult *Pleurobrachia* (PpiSox2/12, part of the SoxC group) [35]. With the possible exception of MleSox1 (SoxB group; very light expression in upper comb rows), MleSox4 (SoxE group; expression in comb rows), and MleSox6 (unclassified; light expression in comb rows) which all display some comb row expression in the newly hatched 24 hpf cydippid stage, an interesting finding of this study is the apparent lack of clear *Sox* gene expression in regions of the developing meridional canals/comb rows (especially by the *Mnemiopsis* SoxC gene, unlike what is observed in *Pleurobrachia*), where germ cells presumably arise during the early juvenile stages of *Mnemiopsis*. Although fate mapping experiments have shown the cellular lineage of many adult structures, it has not yet been possible to follow labeled embryos long enough to determine the precise origin of germ cells [30]. Ctenophores are thought to specify their germline during early cydippid stages via inductive cell signaling, from the meridional canal endoderm, but this has not been confirmed experimentally and other origins of the germline are possible [54]. A small percentage of *Mnemiopsis* can spawn for a limited period of time as 7- to 13-day-old cydippids that measure 1.8 to 2.8 mm oral-aboral length [30], which indicates that gonads can be fully developed and functional by this time in some individuals. Adult reproduction begins at 30 mm oral-aboral length, at an age of 13 to 17 days [55]. Repeated attempts to perform *in situ* hybridizations of *Sox* genes on slightly older (4 to 7 days) cydippid juveniles were unsuccessful, as their tissues are very fragile and tend to fall apart. Some traditional germline marker genes (such as *Piwi*) also do not show expression in developing meridional canals/comb rows during the early 24 to 36 hpf cydippid stage in *Mnemiopsis*[56]. Although the timing of germline specification and origin of germ cells in *Mnemiopsis* remains an open question, based on the above gene expression evidence, it seems plausible that germ cells may not yet be specified by 24 hpf in *Mnemiopsis*, when the animals are only 250 to 300 μm in diameter. Further study of MleSox4 (SoxE group) and additional germline marker genes will help to answer open questions regarding germline specification in *Mnemiopsis*.

### Comparison of *Sox* gene expression between ctenophore species

Comparisons between *Mnemiopsis* and *Pleurobrachia* mRNA expression patterns were possible for the juvenile cydippid stage for four sets of *Sox* orthologs (Table 2). There was general correspondence in expression for the group B orthologs (MleSox1 and PpiSox3) in the pharynx, tentacle bulb, and apical sense organ, however, there was very light expression in the uppermost part of the comb row of MleSox1 that was not seen in PpiSox3. The group C orthologs had similar expression in the tentacle bulb and apical organ between the two species, but MleSox2 lacked expression in comb rows that was seen in PpiSox2/12 and the *Pleurobrachia* SoxC genes lacked expression in the pharynx that was seen in MleSox2. Correspondence in expression in the tentacle bulbs, apical sense organ, and pharynx was seen for one set of group E orthologs (MleSox3 and PpiSox1). The second set of group E orthologs (MleSox4 and PpiSox6) exhibited very different expression patterns from one another; MleSox4 had a broad pattern of expression in the tentacle bulbs, apical organ, comb rows, and pharynx at the cydippid stage, while PpiSox6 was expressed exclusively in developing comb rows. Although the expression patterns examined in these two studies each only capture a snapshot in time, the comparisons between the two ctenophore species illustrate how orthologous *Sox* genes likely share many similar functions, while at the same time, developing some species-specific roles during development. Overall, however, the well-established role of the *Sox* family in the maintenance of stem cell pools during development [53] seems to be conserved in ctenophores, at least as much as can be indicated by the zones of cell proliferation seen in both *Mnemiopsis* and *Pleurobrachia* in the apical sense organ, upper tentacle bulbs, and comb rows, which overlap with regions of *Sox* gene expression in both species. As discussed by Jager et al. [35], for most invertebrate *Sox* genes (including the ctenophore *Sox* genes), the precise functions of individual *Sox* genes have not been determined; it remains unknown whether the ancient function of particular *Sox* groups is primarily associated with cell proliferation (stem cells/progenitor cells) or with differentiating cells. Functional studies of the ctenophore *Sox* genes in particular cell lineages as they progress would help to address these issues and aid in connecting expression patterns with function.

### Comparison of ctenophore and sponge *Sox* gene expression

*Sycon* SoxB (SciSoxB) is expressed in the ectoderm and in cruciform cells, which are larval sensory cells that may be involved in photoreception [12]. The *Mnemiopsis* SoxB gene (MleSox1) is similarly expressed in the ectoderm in early developmental stages, and in cells that contribute to the apical sense organ where photoreceptors reside [22]. These results indicate a general pattern of conservation of SoxB gene expression in cells involved in light sensing in early animal lineages. In bilaterians, SoxB genes are broadly involved in neurogenesis and the development and specification of the central nervous system [1,57], and these roles may have first begun to emerge in ctenophores and possibly sponges.

In invertebrate bilaterians, SoxE genes are often involved in gonad development (mesodermal derivatives), while SoxF genes are commonly involved in endoderm formation [57]. In the anthozoan cnidarians *Nematostella* and *Acropora*, SoxE and SoxF genes are similarly associated with endodermal expression [33,34]. Fortunato et al. [12] point out that SciSoxE, SciSox6, SciSoxF1, and SciSoxF2 are expressed in choanocytes and in some mesohyl cells in *Sycon*, which could be used to support the concept of homology of the choanoderm plus the mesohyl with endomesoderm in sponges. *Mnemiopsis* SoxE genes (MleSox3 and MleSox4) and the unclassified MleSox6 gene are similarly expressed in areas that overlap with the endomesoderm, and the *Pleurobrachia* SoxF gene (PpiSox8) is endodermally expressed, indicating the importance of these groups of *Sox* genes in endomesoderm specification in the earliest animal lineages.

## Conclusions

Our results support the scenario that true *Sox* family genes arose at the base of the animals and were fully diversified into four of the five well-characterized *Sox* groups (B, C, E, and F) in ctenophores (Figure 7). The phylogeny that places ctenophores as the earliest-branching animal lineage [15-17] provides a framework for understanding the potential loss of SoxF group genes in Porifera and Placozoa and for studying the functions of important developmental genes in the earliest animal lineages (Figure 7). Alternative animal phylogenies that place Porifera or Placozoa as the earliest branch would only alter the interpretation of when the SoxF group arose. The expression patterns generated for five *Mnemiopsis Sox* genes, combined with the regions of cell proliferation indicated by the EdU labeling experiments (which largely overlap with stem cell/progenitor regions in *Pleurobrachia*), are consistent with the established role of *Sox* family genes in the maintenance of stem cell pools. Comparisons between *Mnemiopsis* and *Pleurobrachia Sox* expression patterns during the juvenile cydippid phase highlight the power of using multiple species from the same phylum to understand the evolution of developmental genes within a given phylum. Importantly, our results, interpreted within the framework of the phylogeny that places the Ctenophora lineage at the base of all animals, is consistent with the hypothesis that the ancient primary function of *Sox* family genes was in regulating stem cell maintenance.

## Abbreviations

HMG: High mobility group; NBT: Nitro-blue tetrazolium; BCIP: 5-bromo-4-chloro-3-indolyl phosphate.

## Competing interests

The authors declare that they have no competing interests.

## Authors’ contributions

CS helped conceptualize and design the study, performed the genome searches and the phylogenetic analyses, analyzed and interpreted data, and drafted the manuscript. DS isolated and cloned the genes, performed the expression analyses, analyzed and interpreted data, and aided in manuscript preparation. KP performed the EdU labeling experiment, and analyzed and interpreted data. MQM helped conceptualize and design the study and aided in manuscript preparation. ADB helped conceptualize and design the study and helped in manuscript preparation. All authors read and approved the final manuscript.

## Supplementary Material

Additional file 1 Multiple sequence alignment of HMG-box domain sequences used for phylogenetic analyses in FASTA format. Click here for file

Additional file 2: Figure S1 Phylogenetic tree of *Sox* sequences according to the Bayesian analysis. Species name abbreviations are as in Figure 1. Anthozoan cnidarian sequences are indicated in pink, hydrozoan cnidarian sequences are in orange, placozoan sequences are in purple, poriferan sequences are in green, ctenophoran sequences are in blue, and bilaterian sequences are in black. Bayesian posterior probabilities are shown as colored circles at nodes. Red circles indicate 100% support, blue circles indicate >95% support, and black circles indicate >65% support. Click here for file
