# Supplementary material for: Expression of multiple Sox genes through embryonic development in the ctenophore Mnemiopsis leidyi is spatially restricted to zones of cell proliferation
Source: EvoDevo. 2014 Apr 24;5:15. doi: 10.1186/2041-9139-5-15 (PMC4021642; doi:10.1186/2041-9139-5-15)
Supplement: Additional file 2: Figure S1 — Phylogenetic tree of Sox sequences according to the Bayesian analysis. Species name abbreviations are as in Figure 1. Anthozoan cnidarian sequences are indicated in pink, hydrozoan cnidarian sequences are in orange, placozoan sequences are in purple, poriferan sequences are in green, ctenophoran sequences are in blue, and bilaterian sequences are in black. Bayesian posterior probabilities are shown as colored circles at nodes. Red circles indicate 100% support, blue circles indicate >95% support, and black circles indicate >65% support. [file 2041-9139-5-15-S2.pdf]

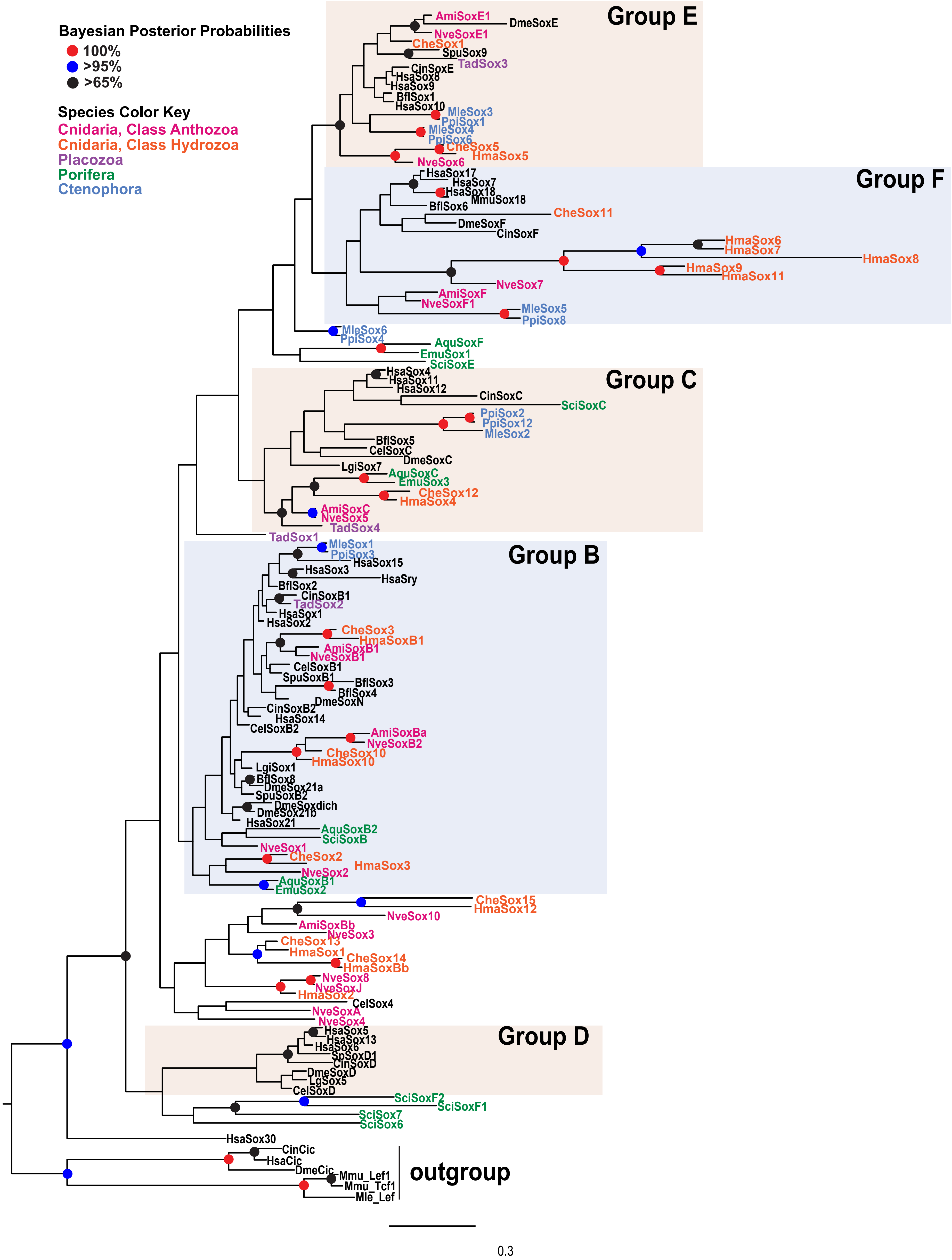
Bayesian Posterior Probabilities
100%
>95%
>65%
Species Color Key
Cnidaria, Class Anthozoa
Cnidaria, Class Hydrozoa
Placozoa
Porifera
Ctenophora
Group E
AmiSoxE1
DmeSoxE
NveSoxE1
CheSox1
SpuSox9
TadSox3
CinSoxE
HsaSox8
HsaSox9
BflSox1
HsaSox10
MleSox3
PpiSox1
MleSox4
PpiSox6
CheSox5
HmaSox5
NveSox6
Group F
HsaSox17
HsaSox7
HsaSox18
MmuSox18
BflSox6
CheSox11
DmeSoxF
CinSoxF
HmaSox6
HmaSox7
HmaSox8
HmaSox9
HmaSox11
NveSox7
AmiSoxF
NveSoxF1
MleSox5
PpiSox8
MleSox6
PpiSox4
AquSoxF
EmuSox1
SciSoxE
Group C
HsaSox4
HsaSox11
HsaSox12
CinSoxC
SciSoxC
PpiSox2
PpiSox12
MleSox2
BflSox5
CelSoxC
DmeSoxC
LgiSox7
AquSoxC
EmuSox3
CheSox12
HmaSox4
AmiSoxC
NveSox5
TadSox4
TadSox1
Group B
MleSox1
PpiSox3
HsaSox15
HsaSox3
HsaSry
BflSox2
CinSoxB1
TadSox2
HsaSox1
HsaSox2
CheSox3
HmaSoxB1
AmiSoxB1
NveSoxB1
CelSoxB1
SpuSoxB1
BflSox3
BflSox4
DmeSoxN
CinSoxB2
HsaSox14
CelSoxB2
AmiSoxBa
NveSoxB2
CheSox10
HmaSox10
LgiSox1
BflSox8
DmeSox21a
SpuSoxB2
DmeSoxdich
DmeSox21b
HsaSox21
AquSoxB2
SciSoxB
NveSox1
CheSox2
HmaSox3
NveSox2
AquSoxB1
EmuSox2
CheSox15
HmaSox12
NveSox10
AmiSoxBb
NveSox3
CheSox13
HmaSox1
CheSox14
HmaSoxBb
NveSox8
NveSoxJ
HmaSox2
CelSox4
NveSoxA
NveSox4
Group D
HsaSox5
HsaSox13
HsaSox6
SpSoxD1
CinSoxD
DmeSoxD
LgSox5
CelSoxD
SciSoxF2
SciSoxF1
SciSox7
SciSox6
HsaSox30
CinCic
HsaCic
DmeCic
Mmu_Lef1
Mmu_Tcf1
Mle_Lef
outgroup
0.3
